# Complete chloroplast genome of *Lens lamottei* reveals intraspecies variation among with *Lens culinaris*

**DOI:** 10.1038/s41598-023-41287-y

**Published:** 2023-09-11

**Authors:** Selda Kurt, Yasin Kaymaz, Duygu Ateş, Muhammed Bahattin Tanyolaç

**Affiliations:** Faculty of Engineering, Department of Bioengineering, Ege University, Izmir, Turkey

**Keywords:** Genome informatics, Phylogeny

## Abstract

*Lens lamottei* is a member of the Fabaceae family and the second gene pool of the genus *Lens*. The environmental factors that drove the divergence among wild and cultivated species have been studied extensively. Recent research has focused on genomic signatures associated with various phenotypes with the acceleration of next-generation techniques in molecular profiling. Therefore, in this study, we provide the complete sequence of the chloroplast genome sequence in the wild *Lens* species *L. lamottei* with a deep coverage of 713 × next-generation sequencing (NGS) data for the first time. Compared to the cultivated species, *Lens culinaris*, we identified synonymous, and nonsynonymous changes in the protein-coding regions of the genes *ndh*B, *ndh*F, *ndh*H, *pet*A, *rpo*A, *rpo*C2, *rps*3, and *ycf*2 in *L. lamottei*. Phylogenetic analysis of chloroplast genomes of various plants under Leguminosae revealed that *L. lamottei* and *L. culinaris* are closest to one another than to other species. The complete chloroplast genome of *L. lamottei* also allowed us to reanalyze previously published transcriptomic data, which showed high levels of gene expression for ATP-synthase, rubisco, and photosystem genes. Overall, this study provides a deeper insight into the diversity of *Lens* species and the agricultural importance of these plants through their chloroplast genomes.

## Introduction

Lentils are nutritionally important protein-rich crops that provide essential and nonessential amino acids^1^, carbohydrates, lipids, dietary fiber, minerals, and water-soluble vitamins^2,3^. Lentils are self-pollinating legumes belonging to Fabaceae (Leguminosae), the third-largest plant family^4^. This family includes plants with economic value^5^. The genus *Lens* consists of seven species belonging to four gene pools. *Lens culinaris* is the cultivated form, while *Lens lamottei*, *Lens orientalis*, *Lens tomentosus*, *Lens ervoides*, *Lens odemensis,* and *Lens nigricans* are wild species^6^. Their natural habitats are distributed in the Mediterranean region and Central Asia^7^.

*Lens lamottei* belongs to the second gene pool of *Lens* and is a member of the Papillionodeae subfamily^3^. Czefronova and colleagues were the first to describe *L. lamottei*, which originated from Spain, Italy, and Turkey^8^. *L. lamottei* has a small seed, at approximately 1.92 mm, with a round shape^9^. Its stipules are horizontal, and the leaves are less dentate^9^. *L. lamottei* has one of the highest average harvest indices, the highest number of seeds per plant, the highest seed yield, and the highest biomass^10^. It is highly resistant to various biotic stressors, such as Stremphylium blight, Fusarium wilt, and Bruchid weevils. It ranks second among wild species in terms of resistance to anthracnose and Ascochyta blight^11^. It also has enhanced tolerance to drought, salinity, and alkalinity^9^.

The chloroplast (cp) is a crucial organelle that plays a major role in photosynthesis, starch production, and metabolism of amino acids, fatty acids, pigments, vitamins, sulfur, and nitrogen^12,13^. It harnesses energy that can be supplied to the plant cell^14^. Cp-based studies are vital in plant biotechnology, especially in plant breeding studies^15^. Cp genomes can be used to construct phylogenetic trees to elucidate distinctive plant features, such as maternal inheritance, high copy numbers, and self-replication ability^16,17^. It provides valuable information about the structural variations and exposes the molecular divergence of related species^18^.

The cp has an independent genome that is around 120–170 kb in size and consists of 120–130 genes mainly involved in photosynthesis^19^. The recently published cp genome of *L. ervoides*, one of the close relatives of *L. lamottei*, is also approximately 120 kb^20^. Cp genome size is more conversed across species as opposed to the mitochondrial genome^21^. It is stable and highly conserved, with the mutation rate of the cp genome lower than the nuclear genome^13,22^. The cp genome has a quadripartite structure with a small single copy region (SSC), a large single copy region (LSC), and two inverted repeat regions (IRs)^22^. Species carrying a single copy IR region in their cp genome structure are classified under the Inverted Repeat Lacking Clade (IRLC)^23^, which includes *Cicer arietinum*, *Pisum sativum*, *Vicia fab*a, *L. culinaris*, and *L. ervoides*^20,24^. The IRLC is a useful model system for examining how the evolution of the plastome is impacted by the loss of a single IR region^25^.

Divergence patterns, substitution rates, and phylogenetic relations across land plants depend heavily on the comparative analyses of their genomic sequences^26,27^. Expansion or contraction of simple sequence repeats (SSRs) can be potential DNA markers for identification of species^18^. Genomic data is also utilized in studying gene expression and regulation. Recently, comparisons of transcriptomic profiles have been reported to determine differential gene expression^28^. Likewise, RNA editing sites and codon usage bias have been studied to identify the role of cp genes and these gene’s regulatory mechanisms^29,30^.

The lack of a cp genome sequence for *L. lamottei* limits its phylogenetic examination across related species and its potential to associate with wild-type features that can be agriculturally beneficial. In the current study, we report the cp sequence of *L. lamottei* and compare it with *L. culinaris*.

## Results

### Genomic features and organization of* L. lamottei* cp DNA

**Figure 1:**
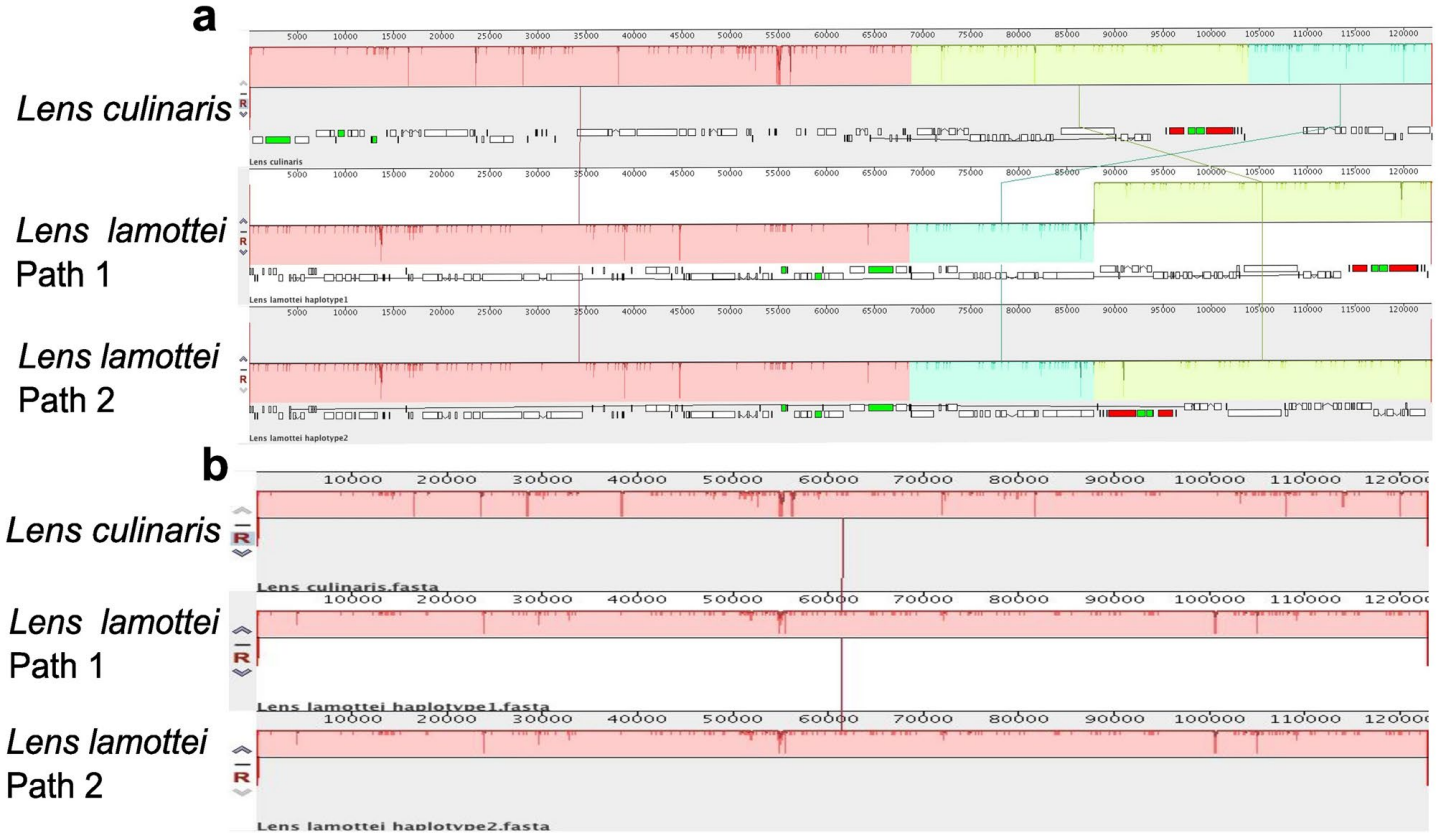
Gene arrangement analysis using Locally Colinear Blocks (LCBs). MAUVE software was used to compare the gene order of *L. lamottei* with the genome of *L. culinari*s cp as a reference. (**a**) Comparison of two possible haplotypes of *L. lamottei* without manual correction. (**b**) Comparison of two possible haplotypes of *L. lamottei* after manual correction.

The BGISEQ-500 platform was used to sequence the cp genome of *L. lamottei*. The de novo cp genome of *L. lamottei* was assembled using the GetOrganelle pipeline via two different assembly paths. To examine genome rearrangement, the Mauve aligner was used to align *L. culinaris* and *L. lamottei*’s cp genome. The alignment calculated the locally collinear blocks (LCB), which represent highly similar, or conserved regions. When these assemblies were compared, certain regions of assembly path 1 were detected to be inverted without any missing parts in reference to *L. culinaris* (Fig. 1a). On the other hand, assembly path 2 was performed by carrying the swapped blocks on the same strand. After manually reorienting the major collinear blocks for both of these assembly options, we found that the *L. lamottei* genome has the same structures and gene content as the cp genome of *L. culinaris* (Fig. 1b).

**Figure 2:**
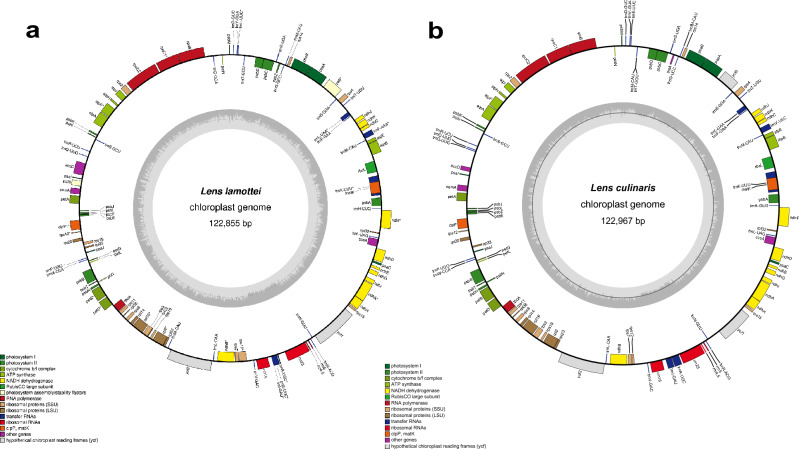
The circular cp genome map of (**a**) *L. lamottei* (**b**) *L.culinaris*. Genes inside the circle are transcribed in the clockwise direction, whereas genes outside the circle are transcribed in the counterclockwise direction. The darker gray on the inside refers to GC content, while the lighter gray on the outside corresponds to AT content. Genes are color-coded according to their function.

As a result of our de novo genome construction analysis, the cp genome of *L. lamottei* was assembled with a size of 122,855 bp and includes the LSC, SSC, and one IR region. Therefore, *L. lamottei* can be classified under the IRLC. Cross-species plastome comparison was done to identify genic regions, including protein-coding, tRNA, and rRNA loci. Figure 2 shows the structural organization of both *Lens* cp genomes with the same orientation of the genic regions. The *L. lamottei* and *L. culinaris* cp genomes were similar in genic content and structural organization. However, due to ambiguities in the annotation approaches, a few genes detected in *L. lamottei* were not found in *L. culinaris*. These genes are *paf*II(*ycf*4), *rps*18, and *rpl*22. Another difference is that the *paf*I gene was annotated as a protein-coding gene in *L. lamottei* but was defined as a pseudogene in the annotation of *L. culinaris*. This is because the annotation of the *L. culinaris* genome was performed using a different tool (DOGMA). When the cp genome of *L. culinaris* was reannotated using the GeSeq tool, the two cp genomes contained the same set of genes. *L. lamottei* and *L. culinaris* have 108 and 105 genes, respectively, that make up the cp genome, including 77, and 73 protein-coding genes, respectively. They have the same number of tRNA (27) and rRNA (4) genes (Supplementary Table S1). These genes have various functions in the cp, including photosynthesis, transcription, and translation (Supplementary Table S2).

Introns play a crucial role in regulating gene expression. Overall, there are 17 intron-containing genes. Among these, six are tRNA genes (*trn*K-UAA, *trn*F-AAA, *trn*L-UAA, *trn*I-GAU, *trn*E-UUC, and *trn*A-UGC), and ten are protein-coding genes (*rpo*C1, *atp*F, *clp*P1, *pet*B, *pet*D, *rpl*16, *rpl*2, *ndh*B, *rps*12, and *ndh*A). *Paf*I (*ycf*3) is the only gene containing two introns. The longest intron (2423 bp) was found in the *trn*K-UUU gene, which also contained the *mat*K gene. The smallest intron (39 bp) was found in the *trn*A-UGC gene.

### Comparative genome analysis

**Figure 3:**
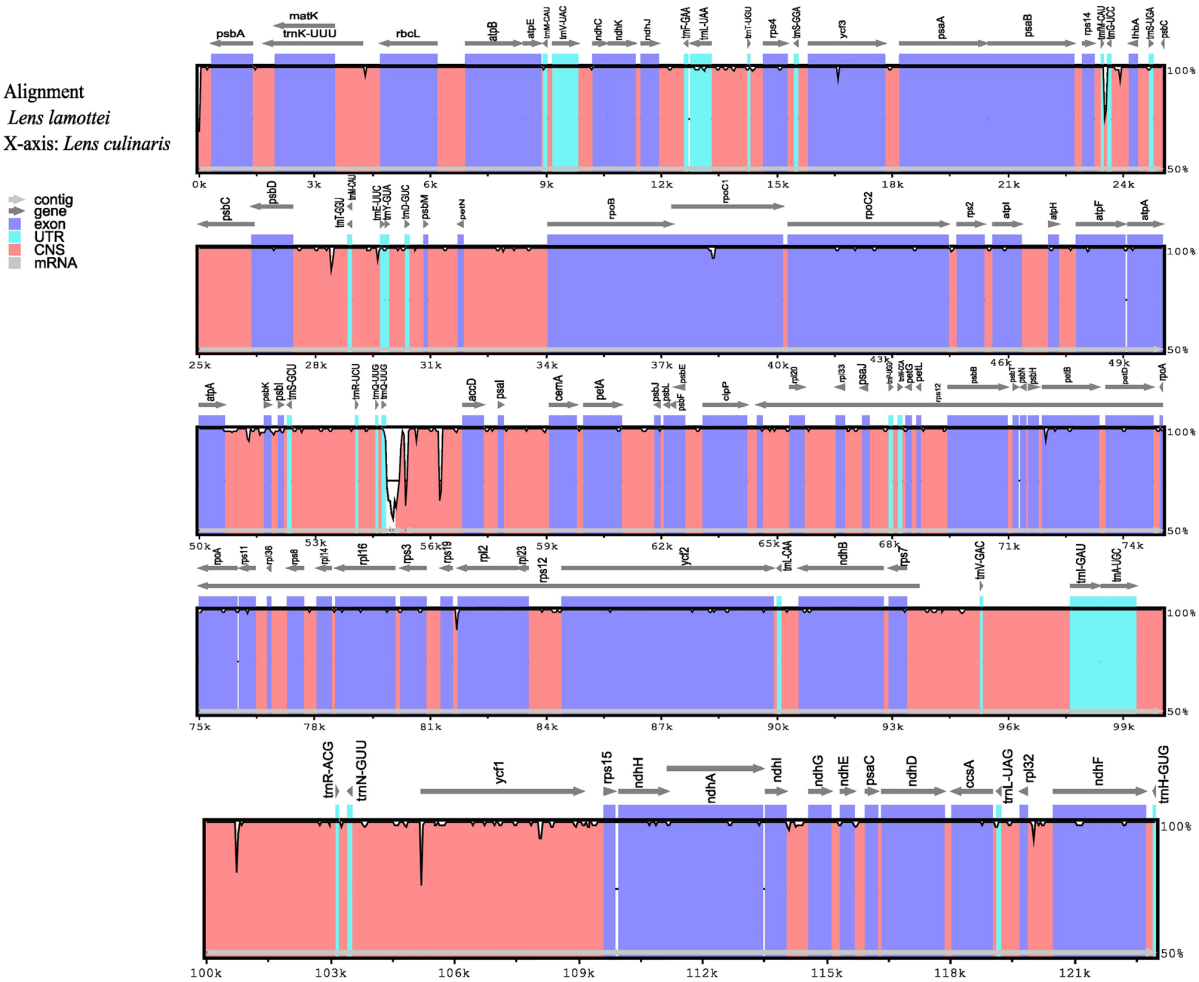
Visualization of alignment of the *L. lamottei* cp genome using *L. culinaris* as a reference sequence using mVISTA. The vertical axis shows the percentage of identity, which goes from 50 to 100%. In the reference plastome, each arrow represents annotated genes and the direction of transcription. Exons are purple, the untranslated region (UTR) is blue, and conserved non-coding sequences (CNS) are pink in the plastome coding areas. A decrease in purple/pink shadowing indicates a decrease in sequence identity (white spaces).

To study the degree of sequence divergence between the cp genomes of *L. lamottei* and *L. culinaris* mVISTA was used to visualize the overall sequence identities of the two cp genomes (Fig. 3). Comparative analyses showed a high sequence similarity with no discernible differences between the wild and cultivated species. We observed similar gene orders and organizations between the two species and highly conserved protein-coding genes with limited substitutions.

**Figure 4:**
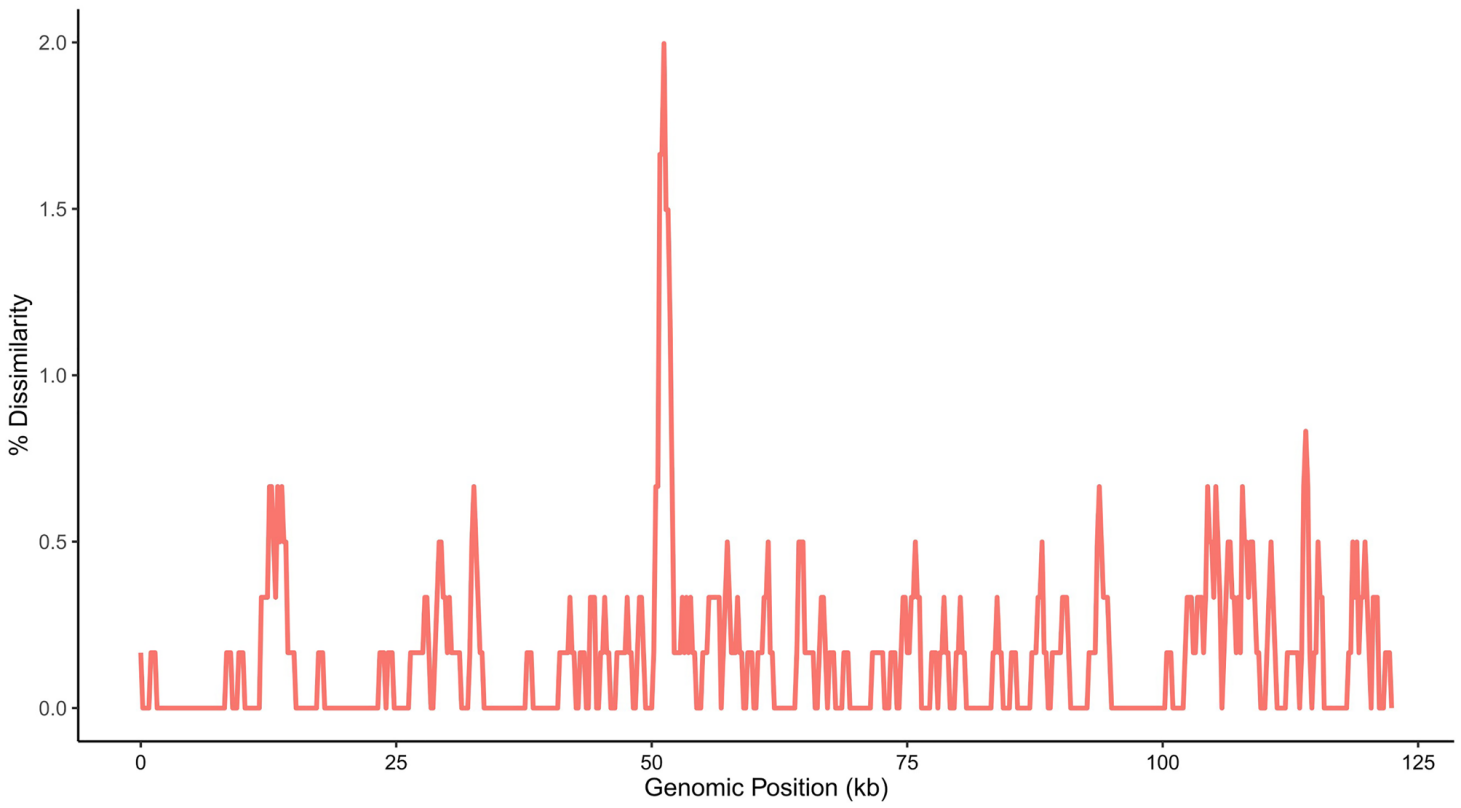
The percent dissimilarity between cp genomes of *L. lamottei* and *L. culinaris* was calculated using a 1 kb window and a 200 bp sliding step. Repetitive regions determined by miropeat are masked.

The hotspot regions in the cp genomes of *L. lamottei* and *L. culinaris* were determined by comparing the nucleotide variation in the coding and noncoding regions. Compared to protein-coding regions, noncoding regions showed higher levels of divergence. Genomic hotspots were detected. High nucleotide variation was found in the intergenic region between the *trnQ*-*accD* genes with a dissimilarity of more than 1.0%. We identified a genomic hotspot in an intergenic region between 50,800 and 52,000 bp (Fig. 4).

### Simple sequence repeats analysis

**Figure 5:**
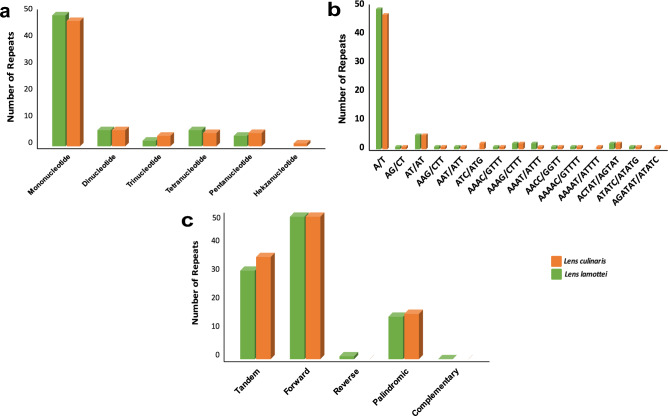
SSRs in *L. lamottei* and *L. culinaris* cp genomes. (**a**) The number of SSRs in the genomes of *L. lamottei* and *L. culinaris.* (**b**) The frequency of SSR motifs is identified in different repeat class types. (**c**)The number of SSR types found in the genomes of *L. lamottei* and *L. culinaris* chloroplasts.

Repetitive region were summarized for both lens species in Fig. 5. SSRs, commonly referred to as microsatellite repeats, are shorter tandem repeats ranging from 1 to 6 bp in length and are found throughout the cp genome^31^. A total of 67 and 68 SSRs, respectively, were found in *L. lamottei* and *L. culinaris* with MISA (MIcroSAtellite Identification Tool). Of these, there were 49 and 47 mononucleotides, 6 dinucleotides in both, 2 and 4 trinucleotides, 6 and 5 tetranucleotides, and 4, and 5 pentanucleotides, respectively (Fig. 5a). One hexanucleotide repeat motif was detected in *L. culinaris* but not in *L. lamottei.* The most common type of SSR was the A/T mononucleotide repeat motif (Fig. 5b). There were differences between the two species in terms of trinucleotide, pentanucleotide, and hexanucleotide motifs. ATC/ATG, AAAAT/ATTTT, and AGATAT/ATATCT 2,1,1 repeat motifs were detected in *L. culinaris*, respectively. However, these motifs were not found in *L. lamottei* (Fig. 5b). Repetitions can be classified under four types: forward, reverse, palindromic, and complementary. Using REPuter, we found 50 forward repeats in both *L. lamottei* and *L. culinaris* cp genomes. There were 15 and 16 palindromic repeats detected in *L. lamottei* and *L. culinaris*, respectively (Fig. 5c). One reverse repeat was found in *L. lamottei* but none in *L. culinaris.* No complementary repeats were found in either species (Fig. 5c). In addition, 31 tandem repeats were found in *L. lamottei* and 36 in *L. culinaris*.

Using *L. culinaris* (NC_027152) as a reference sequence, synonymous and non synonymous variations were compared and evaluated to further investigate the divergence of nucleotides. Variations in protein-coding and non-coding regions in the cp genome were examined. Synonymous and nonsynonymous nucleotide changes in protein-coding regions were detected in the *L. lamottei* genome (Table 1). Out of 73 protein-coding genes, at least one variation was detected in 18 protein-coding genes. Of these, 8 protein-coding genes (*ndh*B, *ndh*F, *ndh*H, *pet*A, *rpo*A, *rpo*C2, *rps*3, and *ycf*2) were found to have nonsynonymous changes, i.e., variation resulting in an amino acid replacement. In particular, more variations (L429F, M1290R, H1324D, F1436L, and I1751M) were identified in the *ycf*2 gene region compared to other genes. In addition, the only synonymous changes occurred in the other 10 genes in the cp genome. In addition, 32 noncoding regions were found (Supplementary Table S3). These genes consist of rRNA and tRNA genes. Only two single nucleotide variations were detected in the *rrn*23 gene.

**Table 1:** Synonymous and nonsynonymous variations in protein-coding genes of *L. lamottei* cp genome.

Gene name	Number of total variation	Number of non synonymous variation	Number of synonymous variations	Nucleotide changes
*atp*A	1	0	1	
*atp*H	1	0	1	
*atp*I	1	0	1	
*ccs*A	1	0	1	
*ndh*A	2	0	2	
*ndh*B	2	1	1	H447Y
*ndh*F	3	1	2	H576D
*ndh*H	2	2	0	I249V, P301A
*pet*A	1	1	0	I297L
*psb*D	1	0	1	
*rpl*16	1	0	1	
*rpo*A	2	1	1	D105N
*rpo*C2	4	2	2	F575L, Q1094E
*rps*11	2	0	2	
*rps*3	1	1	0	F42C
*rps*4	1	0	1	
*rps*7	1	0	1	
*ycf*2	6	5	1	L429F, M1290R, H1324D, F1436L, I1751M

RNA editing is a post-transcriptional modification that results in base conversion. The PREP-Cp (Predictive RNA Editor for plants) program was used to identify putative RNA editing sites in *L. lamottei*. We found 41 predicted RNA editing sites in 17 genes (Supplementary Table S4). The most frequent editing site predicted the change of serine to leucine (%29.3). From nucleotide base C to T, there were all replacement sites. The *ndh*B (7) gene has the highest number of editing sites. At the second codon position, the editing event was most prevalent. In addition, RNA editing sites of *L. culinaris* which were cultivated species of *L. lamottei* were also predicted (Supplementary Table S5), with 41 RNA editing sites in 17 genes and the *ndh*B (7) gene with the highest abundance of sites. The most frequent editing site in *L. culinaris* was also the change of serine to leucine (29.3%).

### Codon preference and expression levels of protein-coding genes

Synonymous codons are multiple codons that encode one amino acid, and synonymous codons typically exhibit varied usage preferences as a result of selection pressure during plant evolution. All 20 amino acid and stop codons in the protein-coding genes of the *L. lamottei*’s cp genome were evaluated for codon content and relative synonymous codon usage (RSCU) values (Supplementary Fig. S1; Supplementary Table S6). A total of 19,509 codons was detected in the cp genome of *L. lamottei*. Looking at the distribution of these codons in amino acids, leucine was the most abundant, while cysteine was the least abundant. Among those amino acids prevalent ATT, which encodes isoleucine (Ile), was the most abundant codon in *L. lamottei*, with 910 TGC, which encodes cysteine (Cys), was the least frequently used codon with 48. Most amino acids show codon biases, while methionine (ATG) and tryptophan (TGG) were expressed by only one codon and have no codon bias. TTA (leucine) had the highest RSCU value, whereas CTC (leucine) had the lowest.

**Figure 6:**
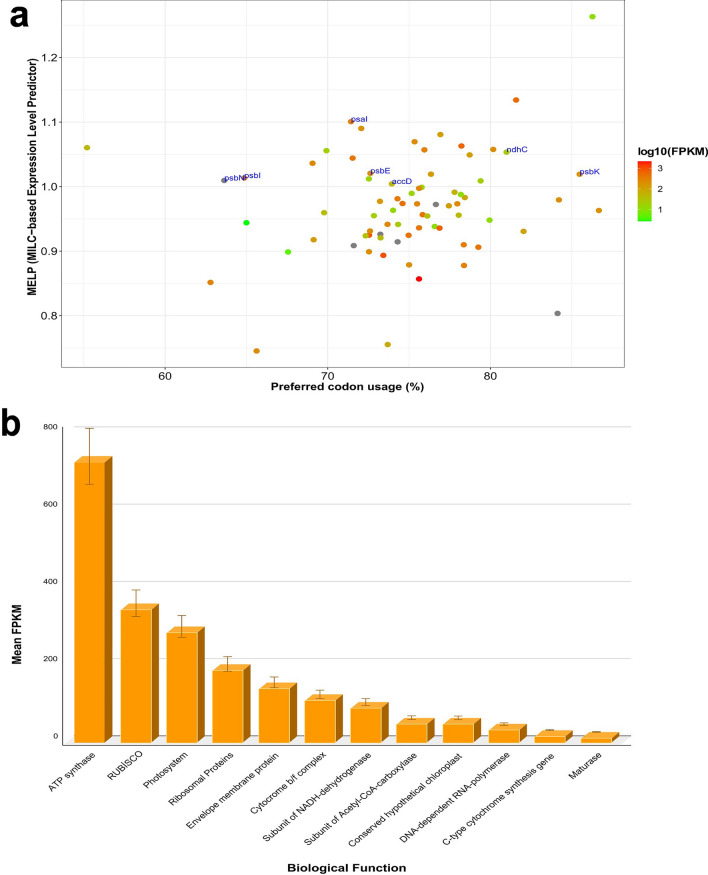
(**a**) The correlation between each protein-coding gene's MELP score and the percentage of codons used that are preferred (RSCU > 1.0). Green indicates ribosomal genes, while red indicates other genes. Gene names are only assigned to non-ribosomal genes with MELP > 1.0. The linear regression is shown by the blue line. (**b**) The value of mean FPKM is used to represent cp gene expression. The X-axis shows the gene in which category they are involved. The Y-axis shows the mean FPKM values of biological function.

Prediction of gene expression based on codon usage requires defining a set of reference genes, which includes all genes encoding ribosomal proteins. Higher MELP (MILC-based Expression Level Predictor) values indicate higher gene expression levels and codon usage bias. The log_10_(FPKM) (Fragments per Kilobase Million) value is expected to increase with the MELP value. Genes with MELP values higher than 1.0 (Fig. 6a) were *psb*N, *psb*I, *psb*E, *psb*K, *psa*I, and *ndh*C, which play roles in photosynthesis, and *acc*D.

Using RNA-seq data from *L. lamottei*, we analyzed the expression of the 76 genes that code for proteins in the cp^32^. Reads were mapped to the *L. lamottei* cp genome we assembled in this study. Taking gene lengths into account, the numbers of reads corresponding to coding genes were computed and normalized. Gene expression values in FPKM were calculated for 76 protein-coding genes (Supplementary Table S7). We found that genes encoding ATP synthase showed the highest expression among all genes (Fig. 6b). This was followed by rubisco and photosystem genes.

### Phylogenetic analysis

**Figure 7:**
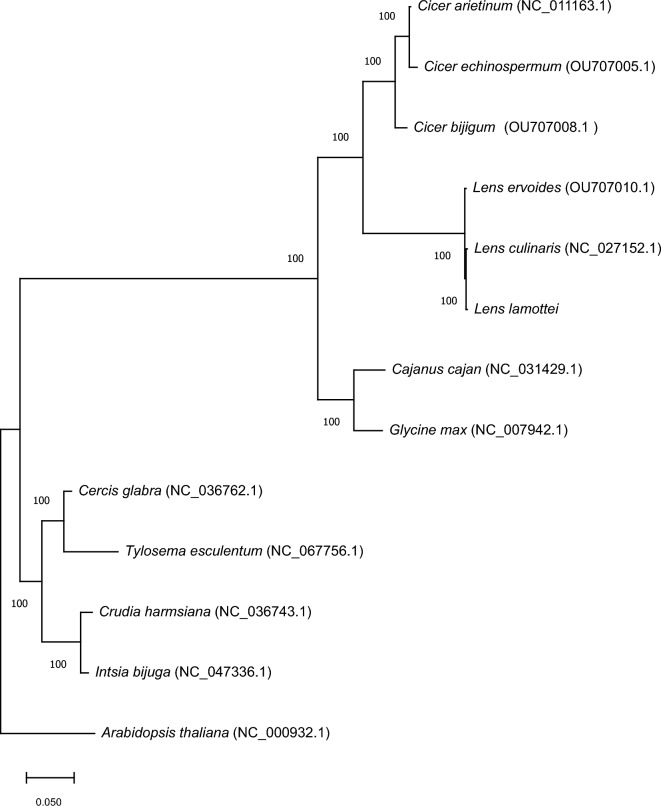
Maximum likelihood phylogenetic tree of 13 whole cp genome which belongs to Leguminosae and *Arabidopsis thaliana* as an outgroup.

In this study, 14 species were included in the phylogenetic analysis to determine the relationships of *L. lamottei* with the other members of the Papilionoideae subfamily (Fig. 7). *Arabidopsis thaliana* was selected as an outgroup. Five species were chosen from Papilionoideae, which belongs to IRLC (*L. culinaris*, *L. ervoides*, *C. ariethinum*, *Cicer echinospermum*, *Cicer bijigum*), and two species from Papilionoideae (*Cajanus cajan*, *Glycine max*), two species from the Detarionoideae subfamily (*Intsia bijuga*, *Crudia hamsiana*), and two species from Cercidoideae subfamily (*Cercis glabra*, *Tylosema esculentum*). Phylogenetic analysis of the 13 cp genomes was performed to provide a better resolution with 1000 bootstraps. In the phylogenetic tree, *L. lamottei* formed a sister clade with *L. culinaris*, indicating that these two species are monophyletic and closely related.

## Discussion

Lentils are richer in protein, carbohydrate, and dietary fiber content compared to other legumes. Therefore, they are consumed at higher rates worldwide due to their importance for human health^1–3^. The cp is a highly conserved organelle, both structurally and genetically, and is involved in many biological functions, particularly in photosynthesis^16,17^. Therefore, the cp genomes of various have been widely sequenced with the development of technologies^22,33^. In comparison to the nuclear genome, the cp genome is inherited maternally and can conveniently be used for phylogenetic investigations due to its short size and conserved structure^34^. We presented the first assembly and annotation of the cp genome of *L. lamottei* and compared it to the cultivated lentil species *L. culinaris*. By examining the genome rearrangements, we found highly similar regions between *L. lamottei* and *L. culinaris*. Two different haplotypes in *L. lamottei* have emerged. Two structural haplotypes of cp genomes that differ in the direction of single-copy regions have been identified in previously published studies^35^. Flip-flop recombination is a plausible theory to explain the existence of structural heteroplasmy and has been reported previously^35,36^. Gene loss, changes in the intergenic region, and expansion, or contraction of the IR region are all variables that affect genome size^37^. The cp genome of *L. lamottei* (122.8 kb) of the Fabaceae family has a similar size to that of the close wild relative *L. ervoides*^20^, the cultivated form *L. culinaris*, and other Fabaceae species such as *O. arctobia*^38^*.* The configuration of cp genomes in the species of this family is similar, with sizes ranging from 107 to 218 kb^39^.

The chloroplast genome of *L. lamottei* has an LSC, SSC region and a single copy IR region. In other plant cp genomes, a pair of IRs are separated by one LSC and one SSC^22^. Some species of the Fabaceae family, including *L. culinaris* contain a single copy of the IR region and belong to the IRLC^26,38^. IRs stabilize the plastome structure and affect its size^40^. However, expansion or contraction, gene loss, and genome rearrangement occurred in the IR regions of some species^41^. Therefore, the cp genomes of *L. lamottei* and *L. culinaris* are highly conserved and similar in genome organization.

The cp gene content was also highly conserved between *L. lamottei* and *L. culinaris*, except for some genes. Some genes annotated in *L. lamottei* could not be identified in the annotation of *L. culinaris*. This could be due to the limitations of DOGMA, the annotation tool used for the reference genome *L. culinaris*. When the cp genome was reannotated using the GeSeq tool, the genes on the cp genome matched with each other. The *L. lamottei* cp genome contains 107 genes, but some genes such as *rps*16 and *inf*A were not identified. Many IRLC species plastomes contain regions with notable changes and rearrangements, such as the loss of introns from the *rps*12 and *clp*P genes and the lack of the *rps*16 gene^42^. The absence of these genes has been reported in other species^20,23^. Introns serve a critical function in gene expression regulation^43^. *L. lamottei* contains 17 genes that have an intron. The absence of one of the *clp*P1 introns in *L. lamottei* is consistent with the loss of the *clp*P1 intron in the genomes of highly conserved papilionoid IRLC members^20,44^.

The sliding window and mVISTA analysis demonstrated high sequence similarity between *L. lamottei* and *L. culinaris*, implying a highly conserved evolutionary model. The intergenic region between the *trn*Q and *acc*D genes has several nucleotide variations in the cp genome of *L. lamottei*. Furthermore, the highly divergent areas were mostly found in the noncoding rather than coding regions, as previously shown in the Fabaceae family^20,45^. These hypervariable intergenic regions can serve as candidate regions for creating genetic markers^46^. These hotspots as markers can be used in the discrimination of species in the *Lens* genus for phylogenetic and identification studies.

Repetitive regions in the genome are essential for genome rearrangements^47^. SSRs can also serve as useful molecular markers for studying genetic diversity and evolutionary relationships in *L. lamottei* and similar species. Nucleotide and tandem repeats in cp genomes are associated with gene duplication, rearrangement, and expansion and can be particularly helpful markers for classifying populations or species^46,48^. SSRs are another type of molecular marker that consists of 1–6 nucleotide repeat units and are commonly employed in population genetics^31^. For both species, mononucleotide SSR is the most prominent motif. Although the numbers of repeat motifs are similar, they differ from each other in some motifs. While *L. culinaris* has one hexanucleotide repeat motif, *L. lamottei* has none. Of these repeat motifs, mononucleotide repeats are the most common SSR motif discovered in the cp genome^49^. Among these mononucleotides, the most common SSR motif found in the *L. lamottei* cp genome is the A/T motif. The common consensus is that plastid SSRs are mostly composed of A/T repeats, with G/C repeats considered as rare^37^. Similar results were found in the wild chickpea species belonging to Fabaceae family and in the *L. ervoides* cp genome^20,50,51^. SSRs have been identified in various organelle genomes and have great application potential for studies on the molecular evolution of plant population genetics and crop breeding^52^.

Understanding the mechanisms underlying molecular evolution critically depends on the estimation of synonymous and nonsynonymous nucleotide mutations^53^. Eight protein-coding genes (*ndh*B, *ndh*F, *ndh*H, *pet*A, *rpo*A, *rpo*C2, *rps*3, and *ycf*2) in the cp genome of *L. lamottei* had nonsynonymous changes. Nonsynonymous mutations are those in which base mutation results in an alteration of the amino acid in the encoded protein^21^. *Ycf*1 and *mat*K genes have been determined as molecular markers due to the detection of nonsynonymous substitutions in these genes in other studies^53,54^. However, in this study, while no substitution occurred in *mat*K and *ycf*1 genes, several mutations were detected for the *ycf*2 gene (in the position of L429F, M1290R, H1324D, F1436L, I1751M). Numerous nonsynonymous changes were detected in the *ycf*2 gene for *L. ervoides*^20^, a close relative of *L. lamottei*. Among noncoding genes, only two single nucleotide substitutions were found in the *rrn*23 gene. Therefore, markers that can be developed using the *ycf2* and *rrn*23 genes may shed light on future studies.

RNA editing is a fundamental post-transcriptional process that commonly occurs in the cp protein-coding regions to restore the evolutionarily conserved amino acid sequence^24,55^. The *ndh*B gene, which had seven RNA editing sites, contained the highest number of editing sites. The NDH protein complex serves an important function in photosynthesis. RNA editing may enhance photosynthetic efficiency and exhibit positive selection during evolution^41^. In 29.3% of the editing sites, serine aminoacids were converted to leucine. Many RNA editing sites can convert the encoded amino acid from polar to nonpolar in both species. In previous studies, unlike hydrophilic mutations, which can affect the secondary structure and function of proteins and improve their genetic information, hydrophobic mutations produce a more stable structure in proteins^24,55,66^. Therefore, the presence of RNA editing sites in the cp genome may elucidate evolutionary mechanisms.

Codon usage significantly influences the evolution of the cp genome and can also be used to determine gene functions, gene expression, and levels of mRNA and proteins^21,56^. The *L. lamottei* cp genome has 19,509 codons, with cysteine having the lowest codon usage and leucine having the highest. In *Coleanthus subtilis*, which also belongs to the legume family, leucine amino acid has the highest codon usage, while cysteine amino acid has the lowest codon usage^56^. In *L. lamottei*, one codon each encodes the amino acids methionine and tryptophan. On the other hand, the remaining amino acids are encoded by two to six codons. Similar codon usage results are observed in wild lentil and chickpea species^20,50,51^. Codon usage could be helpful for phylogenetic association research as codon use can influence the manner of gene mutation^31^.

For cp growth and photosynthesis, appropriate expression of cp genes is essential^57^. Therefore, the expression levels of cp genes in *L. lamottei* were investigated in this study. MELP values of protein-coding genes were obtained using ribosomal genes as reference. In particular, the MELP values of genes involved in photosynthesis were high. In addition, FPKM values were calculated using RNA-seq data to examine the gene expression of cp genes. We found high gene expression levels, especially for the ATP-synthase, rubisco, and photosynthesis-related genes were classified according to their biological functions. Plants synthesize ATP during electron and proton transport. These processes include photosystem I, photosystem II, cytochrome b6/f complex, and ATP synthase^58^. Also, the *rbc*L gene is another gene that is related to the photosystem^59^. Therefore, the expression levels of these genes are expected to be higher. We found that the expression levels of these genes were not positively correlated with the MELP value, which could be due to the highly increased expression of ATP synthase.

To assess the phylogenetic relationships of *L. lamottei*, the complete cp genomes of 14 plant species were used to construct a phylogenetic tree using the Jukes-Cantor model. We used *A. thaliana* as an outgroup to confirm the phylogeny study. For the phylogenetic analysis, the representative species were selected from three main subfamilies of the Fabaceae family: the Papilionoideae, Detarionoideae, and Cercidoideae subfamilies.In this study, results show that *L. lamottei* was closely related to *L. culinaris* and *L. ervoides*. In a previously published study, *L. ervoides* provided the same branching as *L. culinaris*^20^. Thus, these three *Lens* species are expected to be closer to each other than to other species. Therefore, it should present close branching with *L. lamottei*, and cicer species belonging to the IRLC family^24,50,51^. Therefore, as a result of the phylogenetic tree, these two species are expected to be closer to each other than the other species. *C. cajan* and *G. max* are members of the Papilionoideae family^12,24^. The other species are included in the Detarionoideae and Cercidoideae subfamilies^60–63^. Therefore, these species are expected to be more phylogenetically distant from *L. culinaris* in Fig. 7. The strong bootstrap and posterior probability scores in this study suggested that the whole cp genomes could be beneficial in determining the phylogenetic locations and relations of the Papilionoideae tribe for *L. lamottei.*

## Methods

All the methods were carried out in accordance with relevant local/national/international guidelines and regulations.

### Organelle isolation and whole chloroplast genome sequencing of *L. lamottei*

In collaboration with Akdeniz University (Antalya), *L. lamottei* seeds were obtained and genotyped for use in this study. It was registered by the Department of Field Crops herbarium at Akdeniz University, and identified by Prof. Dr. Cengiz Toker. It was deposited with the voucher ID of L. lamottei-01. Rows of seeds were planted in February 2020 and collected from Antalya (Turkey)(36.89722° N, 30.71414° E). Plants were grown at an average temperature of 12 °C. Plants were harvested when their leaves reached the full green stage. For 72 h, leave samples were stored at 4 °C to prevent starch accumulation. 20 g of fresh leaves were harvested and extracted from the cp genome according to the cp DNA isolation method published by Shi et al.^64^. DNA isolation purity was assessed using NanoDrop (Thermo Scientific) and agarose gel electrophoresis.

The cp genomic DNA of the plant was sequenced using the DNA nanoball technique at Beijing Genome Institute (BGI, Hong Kong, China). Briefly, the Agencourt AMPure XP-Medium kit was used. DNA fragments in the 200–400 bp range were chosen after random cpDNA fragmentation and purified the PCR products. With the help of a splint oligosequence, heat-denatured DNAs were converted into single-stranded circular DNA (ssCir DNA) for library preparation. These libraries were sequenced on the BGISEQ-500 platform using whole-genome sequencing. The ssCir DNA molecules were transformed into DNA nanoball (DNB). These DNBs were loaded into nanoarrays with a certain pattern using DNA nanochip technology. Paired-end 100-bp reads were obtained using combinatorial Probe-Anchor Synthesis (cPAS)^65^.

The sequencing qualities were assessed using FastQC^66^ utility. The de novo genome assembly tool GetOrganelle^67^, which was specifically designed for plastid genomes, was used. This pipeline uses the assembly algorithm Spades^68^ to construct contigs and scaffolds from raw sequencing reads. During the assembly process, the algorithm was fed with the *L. culinaris* (NC_027152.1) cp genome as a reference seed and the database of known plastid genomes ('embplant pt'). The constructed scaffolds were visually inspected using Bandage^69^ software to consider possible structural rearrangements. To inspect the assembly quality and read support, raw sequencing reads were aligned back to scaffolds using bowtie2^70^ short read alignment software.

### Gene annotations

The cp genome of *L. culinaris*, which was retrieved from NCBI with accession NC_027152, was used as a reference. MAUVE alignment was performed to analyze gene homology between the two species (*L. lamottei* and *L. culinaris*). Gene order, rearrangements, and structural changes were also identified^71^. The GeSeq^72^ annotation tool was used to determine the locations of protein-coding, tRNA, and rRNA genes. The annotation of tRNA genes was confirmed using tRNAscan-SE v.2.0.7 and ARAGORN v1.2.38^72^. To visualize the cp genome sequence map of *L. lamottei*, Organelle Genomes DRAW (OGDRAW)^73^ was used. The complete cp genome of *L. lamottei* was deposited in the European Nucleotide Archive (ENA) databases with the accession number ERS7635406.

### Comparative genome analysis

Comparison of the cp genomes of the two species (*L. lamottei* and *L. culinaris*) was performed using mVISTA^74^ in Shuffle-LAGAN mode. Using pairwise genome alignment, the divergent areas between the cp genomes of *L. lamottei* and *L. culinaris* were identified and repetitive sequence regions detected by miropeats (version 2.02)^75^ were masked. Custom python scripts were used to determine the sequence single nucleotide substitutions and indels between the two species and their genome-wide hot spots using 1000-bp windows with 200-bp sliding steps. Using the canonical amino acid codon table, synonymous, and nonsynonymous substitutions for the genomic regions were identified. The online PREP-Cp^76^ tool was used to predict RNA editing sites of all protein-coding sequences of *L. lamottei*, with a cutoff value of 0.8.

### Simple sequence repeats analysis

The REPuter^77^ program was used for the detection of the forward, reverse, palindromic, and complementary repeats in the *L. lamottei* cp genome with a minimum repeat size of 30 bp, sequence identity of more than 90%, and hamming distance of 3. Tandem Repeat Finder^78^ was used to identify tandem repeats. MISA^79^ was used for detecting simple sequence repeats within the cp genome. The parameters used were 10 for mononucleotide, 5 for dinucleotide, 4 for trinucleotide, and 3 for tetranucleotide, pentanucleotide, and hexanucleotide.

### Codon usage bias

Functions from the "coRdon" R package (v1.1.3) were used to determine codon usage statistics in the protein-coding regions. Along with the fundamental codon usage frequencies, these statistics also include Measure Independent of Length Composition Value (MILC) and MELP^80,81^. Based on the concept of RSCU, the ratio of observed codon frequency to expected uniform codon usage values was calculated.

### Gene expression analysis

*Lens lamottei* RNA sequencing data were retrieved from NCBI SRA using accession number PRJNA625627. The reads were mapped using STAR^82^ to the assembled cp genome of *L. lamottei*. The featureCounts^83^ program was used to quantify gene expression. FPKM^84^ normalization of expression values for protein-coding genes were calculated.

### Phylogenetic analysis

Following multiple sequence alignment using MAFFT^85^, MEGA 11^86^ was used for the phylogenetic analysis of the aligned sequences. The Jukes–Cantor^87^ model was used to create a maximum likelihood tree for phylogenetic analysis of whole cp genomes of different species. *A. thaliana* was used as an outgroup in the construction of a phylogenetic tree based on the complete cpDNA sequences of 13 species from the Leguminosae subfamilies Papilionoideae (3), IRLC (6), Detarioideae (2), and Cercidoideae (2). Construction and calculation of the phylogenetic tree were performed with 1000 bootstraps. GenBank data of the species used were obtained from the NCBI database. The species that were used were *L. culinaris* (NC_027152.1), *L. ervoides* (OU707010.1), *C. arietinum* (NC_011163.1), *C. echinospermum* (OU707005.1), *C. bijigum* (OU707008.1), *C. cajan* (NC_031429.1), *G. max* (NC_007942.1), *I. bijuga* (NC_047336.1), *C. hamsiana* (NC_036743.1), *C. glabra* (NC_036762.1), *T. esculentum* (NC_067756.1), and *A. thaliana* (NC_000932.1).

### Supplementary Information

Supplementary Information.

## Data Availability

The whole chloroplast genome raw sequence of *L. lamottei* are available in the ENA (European Nucleoide Archive) of EMBL-EBI under the accession number PRJEB47534 and sample identification number ERS7635406.
