# Supplementary material for: Complete chloroplast genome of Lens lamottei reveals intraspecies variation among with Lens culinaris
Source: Sci Rep. 2023 Sep 11;13:14959. doi: 10.1038/s41598-023-41287-y (PMC10495401; doi:10.1038/s41598-023-41287-y)

Complete chloroplast genome of *Lens lamottei* reveals intraspecies variation among with *Lens culinaris*

Selda KURT[1], Yasin KAYMAZ[1], Duygu ATEŞ[1], Muhammed Bahattin TANYOLAÇ[1*]

Supplementary Table S1: Comparison of cp genomes of *L. lamottei* and *L. culinaris*

**Species**	***Lens lamottei***	***Lens culinaris***
Size (bp)	122,855	122,967
Genes	108	105
CDS	77	73
tRNA	27	27
rRNA	4	4
Pseudogene	0	1

Supplementary Table S2: Gene content of *L. lamottei*

Category	Group of genes	Name of genes
Transcription and translation	Large subunit of ribosomal proteins	*rpl*2, *rpl*14, *rpl*16, *rpl*20, *rpl*22*, *rpl*23, *rpl*32, *rpl*33, *rpl*36
	Small subunit of ribosomal proteins	*rps*2, *rps*3, *rps*4, *rps*7, *rps*8, *rps*11, *rps*12, *rps*14, *rps*15, *rps*18*,*rps*19
	DNA-dependent RNA polymerase	*rpo*A, *rpo*B, *rpo*C1, *rpo*C2
	Ribosomal RNA genes	*rrn*4.5, *rrn*5, *rrn*16, *rrn*23
	Transfer RNA genes	*trn*H-GUG, *trn*K-UUU, *trn*M-CAU, *trn*T-GGU, *trn*T-UGU, *trn*V-GAC, *trn*F-GAA,*trn*F-AAA
		*trn*L-UAA, *trn*L-CAA, *trn*L-UAG, *trn*S-UGA, *trn*S-GCU, *trn*S-GGA,
		*trn*G-GCC, *trn*E-UUC, *trn*Y-GUA, *trn*D-GUC, *trn*C-GCA, *trn*R-UCU
		*trn*R-ACG, *trn*Q-UUG, *trn*W-CCA, *trn*P-UGG, *trn*I-GAU, *trn*A-UGC, *trn*N-GUU
Genes for photosynthesis	Photosystem I	*psa*A, *psa*B, *psa*C, *psa*I, *psa*J
	Photosystem II	*psb*A, *psb*B, *psb*C, *psb*D, *psb*E, *psb*F, *psb*H, *psb*I, *psb*J, *psb*K, *psb*L,*psb*M,*pbf*1(*psb*N), *psb*T, *psb*Z(*Ihb*A)
	RUBISCO	*rbc*L
	Subunits of ATPsynthase	*atp*A, *atp*B, *atp*E, *atp*F, *atp*H, *atp*I
	Subunit of NADH- dehidrogenase	*ndh*A, *ndh*B, *ndh*C, *ndh*D, *ndh*E, *ndh*F, *ndh*G, *ndh*H, *ndh*I, *ndh*J, *ndh*K
	Cytochrome b/f complex	*pet*A, *pet*B, *pet*D, *pet*G, *pet*L, *pet*N
Other genes	Protease	*clp*P1
	Maturase	*mat*K
	Envelope membrane protein	*cem*A
	C-type cytochrome synthesis gene	*ccs*A
	Subunit of Acetyl-CoA-carboxylase	*acc*D
	Conserved open reading frames	*ycf*1, *ycf*2, *paf*I(*ycf*3), *paf*II(*ycf*4)*

*Genes found in *L. lamottei* but not in *L. culinaris*.

Supplementary Table S3. Single nucleotide variation of non-coding genes

Gene	Total Variation	Single Nucleotide Variation
*rrn*16	0	
*rrn*23	2	-1256AATAACCACGCGAGCAGGGTAGGACGAAAAGAT,G1363A
*rrn*4.5	0	
*rrn*5	0	
*trn*A-UGC	0	
*trn*D-GUC	0	
*trn*E-UUC	0	
*trn*F-GAA	0	
*trn*G-UCC	0	
*trn*H-GUG	0	
*trn*I-GAU	0	
*trn*K-UUU	0	
*trn*L-CAA	0	
*trn*L-UAA	0	
*trn*L-UAG	0	
*trn*M-CAU	0	
*trn*N-GUU	0	
*trn*P-GGG	0	
*trn*P-UGG	0	
*trn*Q-UUG	0	
*trn*R-ACG	0	
*trn*R-UCU	0	
*trn*S-GCU	0	
*trn*S-GGA	0	
*trn*S-UGA	0	
*trn*T-GGU	0	
*trn*T-UGU	0	
*trn*V-GAC	0	
*trn*V-UAC	0	
*trn*W-CCA	0	
*trn*Y-GUA	0	
*trn*fM-CAU	0	

Supplementary Table S4: RNA Editing Sites of *L. lamottei*

Gene	Nucleotide Position	Aminoacid Position	Effect	Score	Gene	Nucleotide Position	Aminoacid Position	Effect	Score
atpA	791	264	CCC(P)=>CTC(L)	1	*ndhF*	13	5	CAT(H)=>TAT(Y)	1
atpF	107	36	GCA(L)=>GTA(V)	1		241	81	CTT(L)=>TTT(F)	1
ccsA	514	175	CTT(L)=>TTT(F)	0.86		290	97	TCA(S)=>TTA(L)	1
clpP	313	105	CTT(L)=>TTT(F)	1		1,172	391	GCC(A)=>GTC(V)	0.8
matK	991	331	CTT(L)=>TTT(F)	0.86	*ndhG*	166	56	CAT(H)=>TAT(Y)	0.8
	1190	397	TCA(S)=>TTA(L)	0.86		314	105	ACA(T)=>ATA(I)	0.8
ndhA	40	14	CTT(L)=>TTT(F)	1		385	129	CCA(P)=>TCA(S)	0.8
	137	46	GCA(L)=>GTA(V)	1	*petB*	623	208	CCA(P)=>CTA(L)	1
	1085	362	TCT(S)=>TTT(F)	1	*psbF*	77	26	TCT(S)=>TTT(F)	1
ndhB	95	32	TCA(S)=>TTA(L)	1	*rpl2*	262	88	CAC(H)=>TAC(Y)	1
	532	178	CAT(H)=>TAT(Y)	1	*rpo*B	551	184	TCA(S)=>TTA(L)	1
	557	186	TCA(S)=>TTA(L)	0.8		566	189	TCG(S)=>TTG(L)	1
	683	228	CCA(P)=>CTA(L)	1		718	240	CCT(P)=>TCT(S)	1
	692	231	TCT(S)=>TTT(F)	1		2,000	667	TCT(S)=>TTT(F)	1
	776	259	TCA(S)=>TTA(L)	1		2426	809	TCA(S)=>TTA(L)	0.86
	782	261	TCA(S)=>TTA(L)	1	*rpoC1*	41	14	TCA(S)=>TTA(L)	1
ndhD	380	127	CCT(P)=>CTT(L)	1		203	68	ACT(T)=>ATT(I)	0.86
	671	224	TCA(S)=>TTA(L)	1	*rps2*	182	61	GCT(A)=>GTT(V)	0.86
	1402	468	CTT(L)=>TTT(F)	0.8		248	83	TCA(S)=>TTA(L)	1
ndhF	13	5	CAT(H)=>TAT(Y)	1		607	203	CCG(P)=>TCG(S)	0.86
	241	81	CTT(L)=>TTT(F)	1	*rps14*	80	27	CCA(P)=>CTA(L)	1
	290	97	TCA(S)=>TTA(L)	1					
	1172	391	GCC(A)=>GTC(V)	0.8					

Supplementary Table S5: RNA Editing Sites of *L. culinaris*

Gene	Nucleotide Position	Aminoacid Position	Effect	Score	Gene	Nucleotide Position	Aminoacid Position	Effect	Score
*atp*A	791	264	CCC (P) => CTC (L)	1	*rps*14	80	27	CCA (P) => CTA (L)	1
*atp*F	107	36	GCA (A) => GTA (V)	1		101	34	GCT (A) => GTT (V)	0.86
*ccs*A	514	172	CTT (L) => TTT (F)	0.86	*ndh*F	13	5	CAT (H) => TAT (Y)	1
*clp*P	313	105	CTT (L) => TTT (F)	1		241	81	CTT (L) => TTT (F)	1
*mat*K	991	331	CTT (L) => TTT (F)	0.86		290	97	TCA (S) => TTA (L)	1
	1,190	397	TCA (S) => TTA (L)	0.86		1,172	391	GCC (A) => GTC (V)	0.8
*ndh*A	40	14	CTT (L) => TTT (F)	1	*ndh*G	166	56	CAT (H) => TAT (Y)	0.8
	137	46	GCA (A) => GTA (V)	1		314	105	ACA (T) => ATA (I)	0.8
	1,085	362	TCT (S) => TTT (F)	1		385	129	CCA (P) => TCA (S)	0.8
*ndh*B	95	32	TCA (S) => TTA (L)	1	*pet*B	623	208	CCA (P) => CTA (L)	1
	532	178	CAT (H) => TAT (Y)	1	*psb*F	77	26	TCT (S) => TTT (F)	1
	557	186	TCA (S) => TTA (L)	1	*rpl*2	262	88	CAC (H) => TAC (Y)	1
	683	228	CCA (P) => CTA (L)	1	*rpo*B	551	184	TCA (S) => TTA (L)	1
	692	231	TCT (S) => TTT (F)	1		566	189	TCG (S) => TTG (L)	1
	776	259	TCA (S) => TTA (L)	1		718	240	CCT (P) => TCT (S)	1
	782	261	TCA (S) => TTA (L)	1		2,000	667	TCT (S) => TTT (F)	1
*ndh*D	380	127	CCT (P) => CTT (L)	1		2,426	809	TCA (S) => TTA (L)	0.86
	671	224	TCA (S) => TTA (L)	1	*rpo*C1	41	14	TCA (S) => TTA (L)	1
	1,402	468	CTT (L) => TTT (F)	0.8		203	68	ACT (T) => ATT (I)	0.86
*ndh*F	13	5	CAT (H) => TAT (Y)	1	*rps*2	182	61	GCT (A) => GTT (V)	0.86
	241	81	CTT (L) => TTT (F)	1		248	83	TCA (S) => TTA (L)	1
	290	97	TCA (S) => TTA (L)	1		607	203	CCG (P) => TCG (S)	0.86
	1,172	391	GCC (A) => GTC (V)	0.8					

Supplementary Table S6: Codon usage, RSCU values and encoded amino acids for *L. lamottei*

Codon	Aminoacid	CU_*L. Lamottei*	RSCU_*L. Lamottei*	Aminoacid Name	Codon	Aminoacid	CU_*L. Lamottei*	RSCU_*L. lamottei*	Aminoacid Name
TAA	*	47	1.78	Stop	ATG	M	453	1	Methionine
TAG	*	13	0.49		AAT	N	687	1.57	Asparagine
TGA	*	19	0.72		AAC	N	188	0.43	
GCT	A	541	1.85	Alanine	CCT	P	337	1.66	Prolin
GCC	A	165	0.56		CCC	P	146	0.72	
GCA	A	335	1.14		CCA	P	248	1.22	
GCG	A	130	0.44		CCG	P	83	0.41	
TGT	C	173	1.57	Cysteine	CAA	Q	563	1.6	Glutamine
TGC	C	48	0.43		CAG	Q	140	0.4	
GAT	D	603	1.63	Aspartate	CGT	R	251	1.4	Arginine
GAC	D	138	0.37		CGC	R	74	0.41	
GAA	E	758	1.52	Glutamate	CGA	R	233	1.3	
GAG	E	237	0.48		CGG	R	87	0.49	
TTT	F	842	1.44	Phenylalanine	AGA	R	323	1.8	
TTC	F	327	0.56		AGG	R	108	0.6	
GGT	G	505	1.45	Glycine	TCT	S	436	1.85	Serine
GGC	G	142	0.41		TCC	S	214	0.91	
GGA	G	547	1.57		TCA	S	287	1.22	
GGG	G	199	0.57		TCG	S	109	0.46	
CAT	H	346	1.56	Histidin	AGT	S	294	1.24	
CAC	H	98	0.44		AGC	S	77	0.33	
ATT	I	910	1.54	Isoleucine	ACT	T	419	1.66	Thereonine
ATC	I	287	0.49		ACC	T	172	0.68	
ATA	I	573	0.97		ACA	T	317	1.26	
AAA	K	779	1.56	Lysine	ACG	T	100	0.4	
AAG	K	220	0.44		GTT	V	424	1.54	Valine
TTA	L	734	2.09	Leucine	GTC	V	130	0.47	
TTG	L	416	1.18		GTA	V	413	1.5	
CTT	L	445	1.26		GTG	V	134	0.49	
CTC	L	102	0.29		TGG	W	341	1	Tyrptophan
CTA	L	283	0.8		TAT	Y	586	1.66	Tyrosine
CTG	L	131	0.37		TAC	Y	121	0.34	

Supplementary Table S7: Gene classification of *L. lamottei* and FPKM value of each gene

Gene Name	Group of Genes	FPKM	Total FPKM	Mean FPKM	Gene Name	Group of Genes	FPKM	Total FPKM	Mean FPKM
*atp*A	ATP synthase	417.35	4,343.31	723.88	*ndh*D	Subunit of NADH-dehydrogenase	18.99	969.58	88.14
*atp*I	ATP synthase	502.79			*ndh*F	Subunit of NADH-dehydrogenase	11.17		
*atp*F	ATP synthase	838.92			*ndh*A	Subunit of NADH-dehydrogenase	127.66		
*atp*E	ATP synthase	194.12			*ndh*B	Subunit of NADH-dehydrogenase	39.08		
*atp*H	ATP synthase	2,144.31			*ndh*C	Subunit of NADH-dehydrogenase	23.02		
*atp*B	ATP synthase	245.81			*ndh*E	Subunit of NADH-dehydrogenase	256.92		
*ccs*A	C-type cytochrome synthesis gene	15.72	15.72	15.72	*ndh*G	Subunit of NADH-dehydrogenase	232.60		
*ycf*1	Conserved hypothetical chloroplast	27.17	189.04	47.26	*ndh*H	Subunit of NADH-dehydrogenase	84.71		
*paf*I	Conserved hypothetical chloroplast	63.36			*ndh*I	Subunit of NADH-dehydrogenase	102.07		
*paf*II	Conserved hypothetical chloroplast	93.92			*ndh*J	Subunit of NADH-dehydrogenase	33.24		
*ycf*2	Conserved hypothetical chloroplast	4.60			*ndh*K	Subunit of NADH-dehydrogenase	40.10		
*rpo*B	DNA-dependent RNA-polymerase	24.08	93.48	31.16	*rps*2	Ribosomal Proteins	200.67	3,547.01	186.68
*rpo*C1	DNA-dependent RNA-polymerase	55.72			*rps*4	Ribosomal Proteins	19.51		
rpoC2	DNA-dependent RNA-polymerase	13.67			*rpl*14	Ribosomal Proteins	253.89		
*cem*A	Envelope membrane protein	139.12	139.12	139.12	*rpl*16	Ribosomal Proteins	187.16		
*mat*K	Maturase	10.81	10.81	10.81	*rpl*2	Ribosomal Proteins	413.15		
*psa*A	Photosystem I	445.30	5,959.68	283.79	*rpl*20	Ribosomal Proteins	18.72		
*psa*B	Photosystem I	715.21			*rpl*23	Ribosomal Proteins	111.52		
*psa*C	Photosystem I	389.92			*rpl*32	Ribosomal Proteins	11.54		
*psa*I	Photosystem I	287.68			*rpl*33	Ribosomal Proteins	33.71		
*psa*J	Photosystem I	294.56			*rpl*36	Ribosomal Proteins	566.71		
*pbf*1	Photosystem II	118.62			*rps*11	Ribosomal Proteins	247.85		
*psb*30	Photosystem II	0.00			*rps*12	Ribosomal Proteins	47.18		
*psb*A	Photosystem II	213.59			*rps*14	Ribosomal Proteins	627.31		
*psb*B	Photosystem II	481.42			*rps*15	Ribosomal Proteins	16.63		
*psb*C	Photosystem II	1,004.59			*rps*18	Ribosomal Proteins	22.32		
*psb*D	Photosystem II	0			*rps*19	Ribosomal Proteins	131.11		
*psb*E	Photosystem II	212.25			*rps*3	Ribosomal Proteins	208.48		
*psb*F	Photosystem II	2.74			*rps*7	Ribosomal Proteins	119.21		
*psb*H	Photosystem II	310.87			*rps*8	Ribosomal Proteins	310.34		
*psb*I	Photosystem II	251.90			*pet*A	Cytocrome b/f complex	148.73	645.26	107.54
*psb*J	Photosystem II	43.32			*pet*B	Cytocrome b/f complex	12.40		
*psb*K	Photosystem II	191.00			*pet*D	Cytocrome b/f complex	17.92		
*psb*L	Photosystem II	202.43			*pet*G	Cytocrome b/f complex	79.85		
*psb*M	Photosystem II	371.55			*pet*L	Cytocrome b/f complex	213.13		
*psb*T	Photosystem II	196.80			*pet*N	Cytocrome b/f complex	173.25		
*psb*Z	Photosystem II	225.91			*rbc*L	RUBİSCO	343.74	343.74	343.74
*clp*P1	Protease	65.99	65.99	65.99	*acc*D	Subunit of Acetyl-CoA-carboxylase	47.67	47.67	47.67

Supplemental Figure 1. The 20 amino acid and stop codons in all protein-coding genes of *L. lamottei* chloroplast genome were analyzed for codon content and RSCU value.

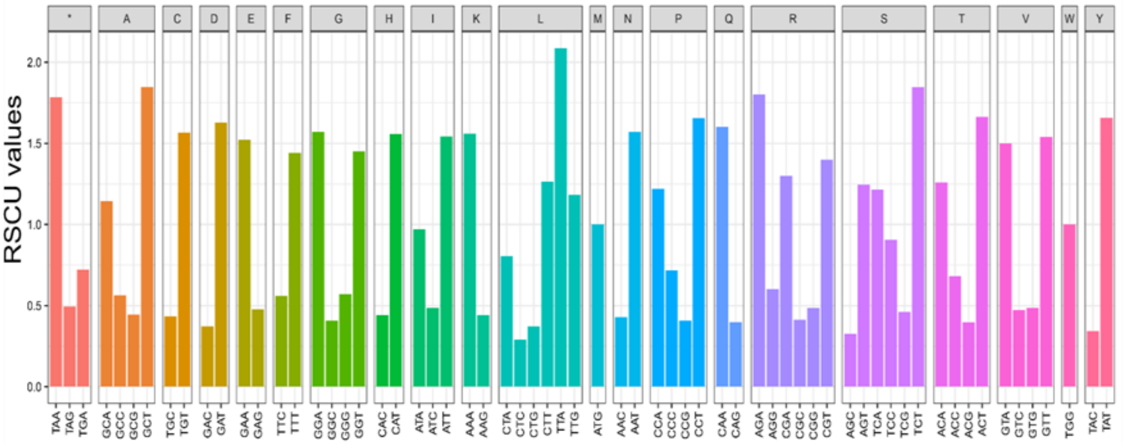

Supplement: Supplementary file 1 — Supplementary Information. [file 41598_2023_41287_MOESM1_ESM.pdf]
